# Improving maternal and neonatal outcomes among pregnant women who are HIV-positive or HIV-negative through the Saving Mothers Giving Life initiative in Uganda: An analysis of population-based mortality surveillance data

**DOI:** 10.1371/journal.pgph.0002801

**Published:** 2024-02-01

**Authors:** Maureen Nabatanzi, Julie R. Harris, Phoebe Namukanja, Steven N. Kabwama, Sandra Nabatanzi, Phoebe Nabunya, Benon Kwesiga, Alex R. Ario, Patrick Komakech

**Affiliations:** 1 Uganda Public Health Fellowship Program, Ministry of Health, Kampala, Uganda; 2 Division of Global Health Protection, US Centers for Disease Control and Prevention, Kampala, Uganda; 3 Department of Community Health and Behavioral Sciences, Makerere University School of Public Health, Kampala, Uganda; 4 Uganda National Institute of Public Health, Ministry of Health, Kampala, Uganda; 5 Office of Health and HIV, US Agency for International Development, Kampala, Uganda; Asian University for Women, BANGLADESH

## Abstract

HIV infection is associated with poor maternal health outcomes. In 2016, the maternal mortality ratio (MMR) in Uganda was 336/100,000, and the neonatal mortality rate (NMR) was 19/1,000. Saving Mothers, Giving Life (SMGL) was a five-year maternal and neonatal health strengthening initiative launched in 2012 in Uganda. We extracted maternal and neonatal data for 2015–2016 from the initiative’s population-based mortality surveillance system in 123 health facilities in Western Uganda. We collected data on the facilities, HIV status, antiretroviral drug (ARV) use, death, birth weight, delivery type, parity, Apgar scores, and complications. We compared mother and baby outcomes between HIV-positive or HIV-negative, computed risk ratios (RR) for adverse outcomes, and used the chi-square to test for significance in differences observed. Among 116,066 pregnant women who attended and gave birth at SMGL-implementing facilities during 2015–2016, 8,307 (7.7%) were HIV-positive, of whom 7,809 (94%) used antiretroviral drugs (ARVs) at the time of delivery. During birth, 23,993 (21%) women experienced ≥1 complications. Neonate Apgar scores <7 (8.8%) and maternal haemorrhage during birth (1.6%) were the most common outcomes. Overall facility MMR was 258/100,000 and NMR was 7.6/1,000. HIV infection increased risk of maternal death (RR = 3.6, 95% Confidence Interval (CI) = 2.4–5.5), maternal sepsis (RR = 2.1, 95% CI = 1.3–3.3), and infant birth weight <2,500g (RR = 1.2, 95% CI = 1.1–1.3), but was protective against maternal complications (RR = 0.92, 95% CI = 0.87–0.97) and perinatal death (RR = 0.78, 95% CI = 0.68–0.89). Among the HIV-positive, ARV non-use increased risk of maternal death (RR = 15, 95% CI = 7.1–31) and perinatal death (RR = 2.3, 95% CI = 1.6–3.4). SMGL reduced facility MMR and NMR below national rates. HIV-infection was associated with maternal sepsis and death. Failure to use ARVs among women living with HIV increased the risk of maternal and perinatal death. Use of the SMGL approach and complementary interventions that further strengthen HIV care, may continue to reduce MMR and NMR.

## Introduction

In the last 30 years, both maternal and neonatal mortality have been declining globally due to concerted strategies to improve equitable access to quality maternal and newborn health care [1, 2]. In 2015, the maternal mortality rate (MMR) in low-income settings was 482/100,000 (compared with 11/100,000 in high-income settings), while the neonatal mortality rate (NMR) was 30/1,000 live births (compared with 3/1,000 in high-income settings) [3, 4]. There are multiple contributors to these high rates in low-income settings, including limited access to health services around the time of delivery to treat hemorrhage, infections (including HIV), high blood pressure, unsafe abortions, and obstructed labor [5, 6]. Similarly, maternal infections and complications around the time of birth, such as asphyxia and congenital birth defects increase the risk of neonatal death [2]. The link between HIV infection in pregnancy and adverse maternal and neonatal outcomes including maternal anaemia, tuberculosis, miscarriages, still births, preterm births and low birth weight babies is well established [7, 8]. In low-income settings, risk of adverse outcomes among HIV infected pregnant women is further complicated by co-infections such as malaria and micronutrient deficiencies like anaemia [9, 10]. Even in the presence of other effective interventions, untreated HIV infection, and/or low CD4 counts has an independent impact on both maternal and neonatal mortality [7, 11]. Most maternal and newborn deaths can be prevented by improving women’s access to facility-based skilled health services. For pregnant women, these include treatment for hypertension and malaria, drugs and transfusions to manage severe bleeding during childbirth, and the use of antiretroviral drugs (ARVs) for women living with HIV [6]. Essential care for newborns includes thermal protection, umbilical cord care, breastfeeding, preventive ARVs if exposed to HIV, immunization, nutrient supplements, and responding to danger signs like feeding difficulties and breathing problems, reduced activity, fever or convulsions [2, 6]. Despite the availability of many of these interventions in Sub-Saharan Africa, as of 2015, the regional neonatal mortality rate was the highest in the world [4, 5]. The 2016 Uganda demographic health survey reported maternal mortality and neonatal mortality rates of 336 per 100,000 and 19 per 1,000 live births, respectively [12].

To accelerate progress towards reducing MMR and NMR, in 2015, the United Nations set global Sustainable Development Goal targets of MMR at <70/100,000 and NMR at <12/1,000 by 2030 [13]. Countries implemented interventions to achieve these through health financing reforms, antenatal, obstetric, and postnatal care, ARVs for HIV-infected pregnant and postpartum women, and essential newborn care [14–16]. One such intervention was the Saving Mothers, Giving Life (SMGL) initiative, funded by the United States President’s Emergency Plan for AIDS Relief (PEPFAR) and launched in 2012 in Uganda and Zambia to reduce maternal and neonatal mortality [17]. In targeted districts, public and private health networks received support to reduce women’s time to seeking, reaching, and receiving quality maternal, newborn, and HIV health services including HIV testing for pregnant women and provision of ARVs [17]. Within these districts, health centres implementing SMGL ensured that every pregnant woman had access to safe delivery and testing of newborns exposed to HIV. In the event of complications, life-saving emergency obstetric and newborn care was provided within two hours [18]. Provision of HIV services for pregnant women is especially important in countries with high HIV prevalence, such as Uganda, in which the prevalence of HIV among adult women was 7.6% in 2016 and 7.1% in 2020 [19, 20].

In Uganda, SMGL was piloted in 2012 in Kibaale, Kabarole, Kyenjonjo, and Kamwenge Districts in Western Uganda [18, 21]. In addition to obstetric and newborn care during the first year of implementation in Uganda, 82% of SMGL facilities provided HIV testing and antiretroviral drugs (ARVs) for expectant women who were HIV-positive [22]. All SMGL facilities together achieved a 35% reduction in MMR in this first year [21]. In 2014, SMGL was expanded to six districts in Northern Uganda [18]. By the end of the five-year initiative, SMGL had successfully reduced maternal mortality by 44% in target facilities in Uganda [17]. However, it was unclear if both HIV-positive and HIV-negative populations attending SMGL facilities had achieved the same benefits in maternal and perinatal outcomes. We compared maternal and perinatal outcomes and mortality rates among women who were HIV-positive or HIV-negativein SMGL facilities in the four pilot districts of western Uganda during 2015 and 2016 to inform future interventions modelled on the SMGL initiative.

## Methods

### Study site

In Uganda, maternal and perinatal healthcare services are provided based on the level of health facility a woman visits. The lowest level facilities, known as health centre (HC) IIs, provide essential obstetric care, HIV services, and intermittent preventive treatment for malaria, while HC IIIs provide normal and assisted deliveries, basic emergency obstetric care, and first aid for obstetric complications. In addition to the services provided by the lower levels, HC IVs and hospitals provide comprehensive emergency obstetric care such as Cesarean sections and blood transfusions, and regional and national referral hospitals receive referrals for women with high-risk pregnancies [23].

This study utilized secondary data from health facilities that implemented the SMGL initiative in the four pilot districts: Kibaale, Kabarole, Kyenjonjo, and Kamwenge in Western Uganda during 2015–2016. In the four districts, SMGL initiative data used were from 123 health facilities for 2015 and 2016. Pregnant women who attended the SMGL implementing health facilities accessed the maternal and perinatal services and thus participated in the initiative.

### Data source and inclusion criteria

We used routinely-collected data for pregnant women collected between 2015 and 2016 and kept in the SMGL database from the four pilot facilities. All pregnant women who were residents of the districts of the facilities were included in the SMGL initiative. In total, 116,066 pregnant women participated in the initiative. These secondary data were collected using Pregnancy Outcomes Monitoring System, a population-based mortality surveillance system used for the SMGL initiative. The Pregnancy Outcome Monitoring System captured pregnant women and their babies’ data on district, death, birth weight, delivery type, Apgar scores, complications, and ARV use (for pregnant women who are HIV-positive). Apgar scores are used to assess newborns’ physiology immediately after birth and to monitor response to resuscitation [24]. During Apgar scoring, the newborn’s color, heart rate, reflexes, muscle tone, and respiration are assessed for signs of insufficient blood flow and scored. The scores are recorded at 1 minute after birth and afterwards at intervals of 5 minutes; scores between 7 to 10 are considered positive, while scores below 7 are considered low [24].

### Data analysis

The MMR was computed as a ratio of maternal (pregnancy or childbirth-related) deaths occurring within 42 days of delivery to total deliveries per 100,000 [4]. NMR was computed as a proportion of neonatal deaths (live births during the first 28 completed days of life) to total births per 1,000 [3]. Maternal outcomes were monitored up to 42 days after delivery. We analyzed using Stata [25] and described maternal district, age, and parity, HIV prevalence, and ARV use among women and babies as means, medians, and proportions. We computed the proportion of pregnant women who participated in the SMGL initiative at each health facility level, delivery types, and outcomes using Stata and plotted graphs of delivery types using Microsoft Excel. Maternal outcomes (death, hemorrhage, sepsis, malaria, and anemia) and infant outcomes (neonatal death, stillbirth, Apgar scores, and birth weight) were analyzed as proportions.

We compared the risk of adverse maternal and perinatal outcomes among women were HIV-positive and women who were HIV-negative and used the chi-square to test for association. Statistical significance was set at p<0.05.

### Ethics statement

This study used secondary data collected by the SMGL initiative (2014–2016) during routine standard of care activities embedded in the district health care system. These data were accessed on 18 July, 2019 for this study. The data were aggregated with no individual patient identifiers and the authors did not have access to information that could identify individual participants. The study protocol was reviewed and approved locally by the Makerere University Higher Degrees, Research and Ethics committee, as well as the US Centers for Disease Control and Prevention (CDC) through the Science Integrity Branch of the Division of Global HIV and TB in accordance with CDC human research protection procedures and was determined to be non-research. Data were only accessed by the study team. This activity was reviewed by CDC and was conducted consistent with applicable federal law and CDC policy.

§See e.g., 45 C.F.R. part 46, 21 C.F.R. part 56; 42 U.S.C. §241(d); 5 U.S.C. §552a; 44 U.S.C. §3501 et seq.

## Results

### Sites of the SMGL initiative

**Fig 1:**
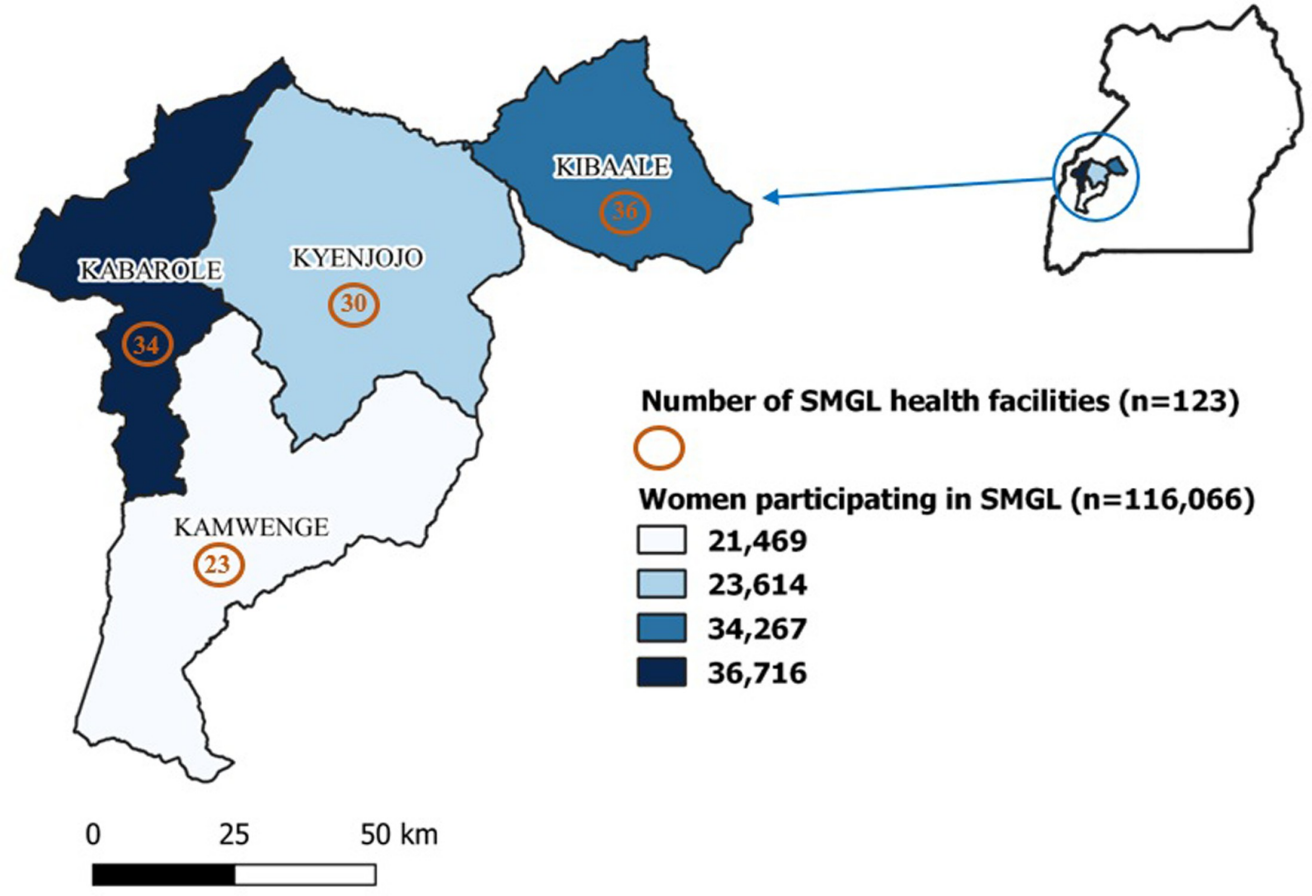
Number of health facilities and pregnant women participating in the SMGL initiative in four districts in Western Uganda, 2015–2016.

Our study focused on 123 health facilities that implemented the SMGL initiative in Kibaale, Kabarole, Kyenjonjo, and Kamwenge Districts in Western Uganda (Fig 1).

**Fig 2:**
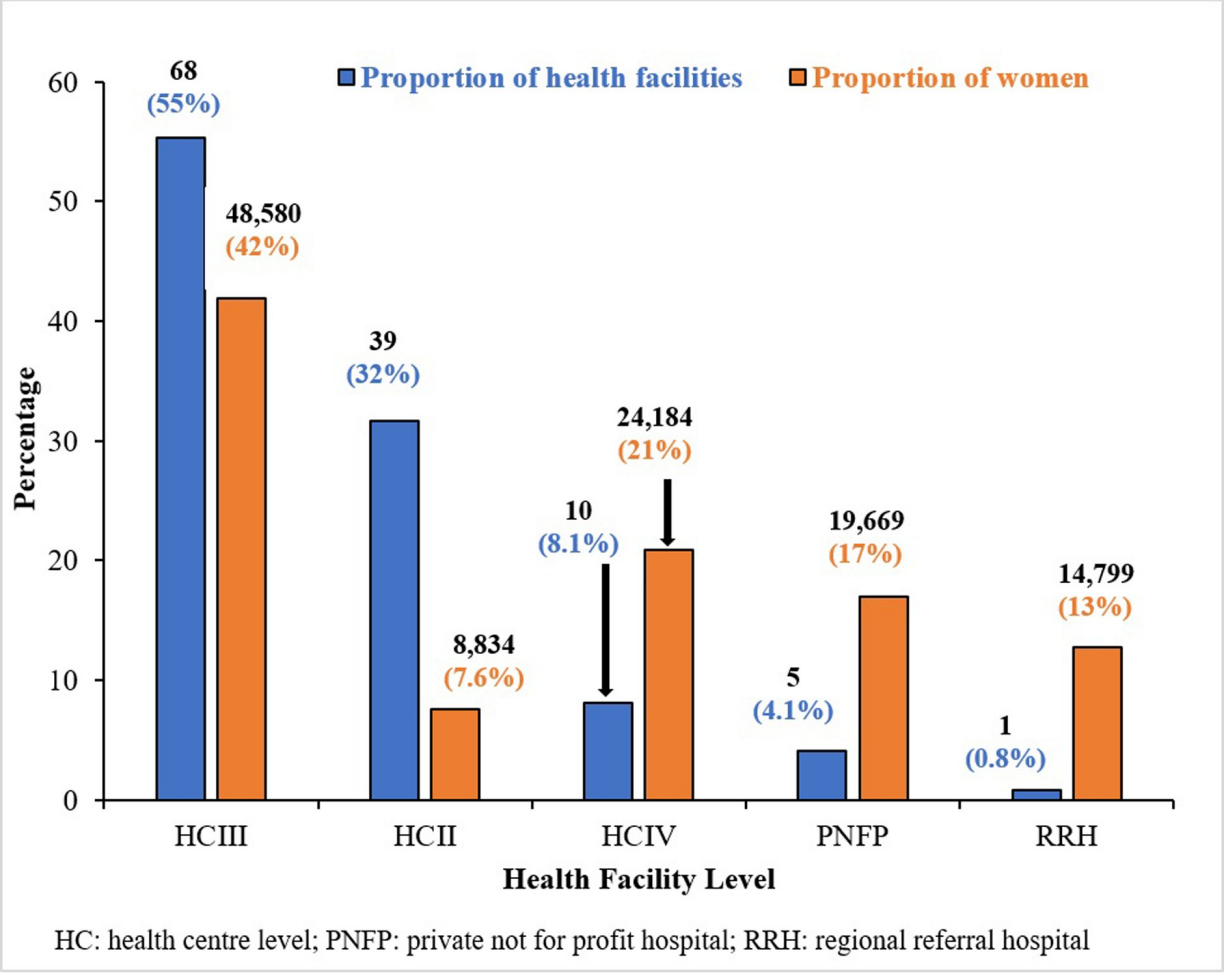
Levels of health facilities and proportion of pregnant women that attended the facilities during the SMGL initiative in Western Uganda, 2015–2016.

Among the four districts, 116,066 pregnant women participated in the SMGL initiative at their facility of attendance (Fig 1). Of the health facilities, 68 (55%) were HC III (Fig 2). Of the 116,066 pregnant women who participated in the SMGL initiative, 48,580 (42%) accessed the services at HC IIIs (Fig 2).

### Characteristics of women and newborns in the SMGL initiative

We abstracted maternal and perinatal data for 116,066 women; the median age was 23 years, mean parity was 2.6 children, and 8,307 (7.2%) were HIV-positive. Among the 8,307 women who were HIV-positive, 7,809 (94%) used ARVs, and 7,173 (86%) of their babies were enrolled on ARVs immediately after birth (Table 1).

**Table 1:** Demographic characteristics of women and babies in the SMGL initiative in four pilot districts in Western Uganda, 2015–2016.

Characteristic	Statistic
Women in SMGL	116,066
Median maternal age	23 (Range = 11–57)
HIV-positive	8,307 (7.2%)
ARV use among HIV-positive	7,809 (94%)
ARV use among babies who were HIV-exposed	7,173 (86%)
Mean parity	2.6 (SD±2.1)*

*SD = Standard Deviation

### Maternal and perinatal outcomes at SMGL-implementing facilities

Among the 116,066 pregnant women at SMGL-implementing facilities, 288 died (MMR = 258/100,000). Haemorrhage (n = 1,891, 1.6%) and sepsis (n = 666, 0.57%) were the most common adverse maternal outcomes. Five hundred and ninety-five (<1%) women had malaria at the time of delivery, and 237 (0.2%) were anaemic. There were 830 neonatal deaths (NMR = 7.6 per 1,000). Low Apgar score (<7) at 1 min (n = 9,494, 8.8%) and low birth weight (<2,500g) (8,218, 7.9%) were the most common neonatal and perinatal adverse outcomes (Table 2).

**Table 2:** Maternal and perinatal or neonatal outcomes among pregnant women in the SMGL initiative in Western Uganda, 2015–2016.

Outcomes (n = 116,066)	n (%)
**Maternal outcome**	
Hemorrhage	1,891 (1.6)
Sepsis	666 (0.57)
Malaria	595 (0.51)
Maternal death	288 (0.26)
Anaemia	237 (0.20)
**Perinatal or Neonatal outcome**	
Low Apgar score at 1min	9,494 (8.8)
Low birth weight	8218 (7.9)
Low Apgar score at 5min	4,911 (4.9)
Fresh still birth	1,740 (1.6)
Macerated still birth	1,339 (1.2)
Neonatal death	830 (0.76)

**Fig 3:**
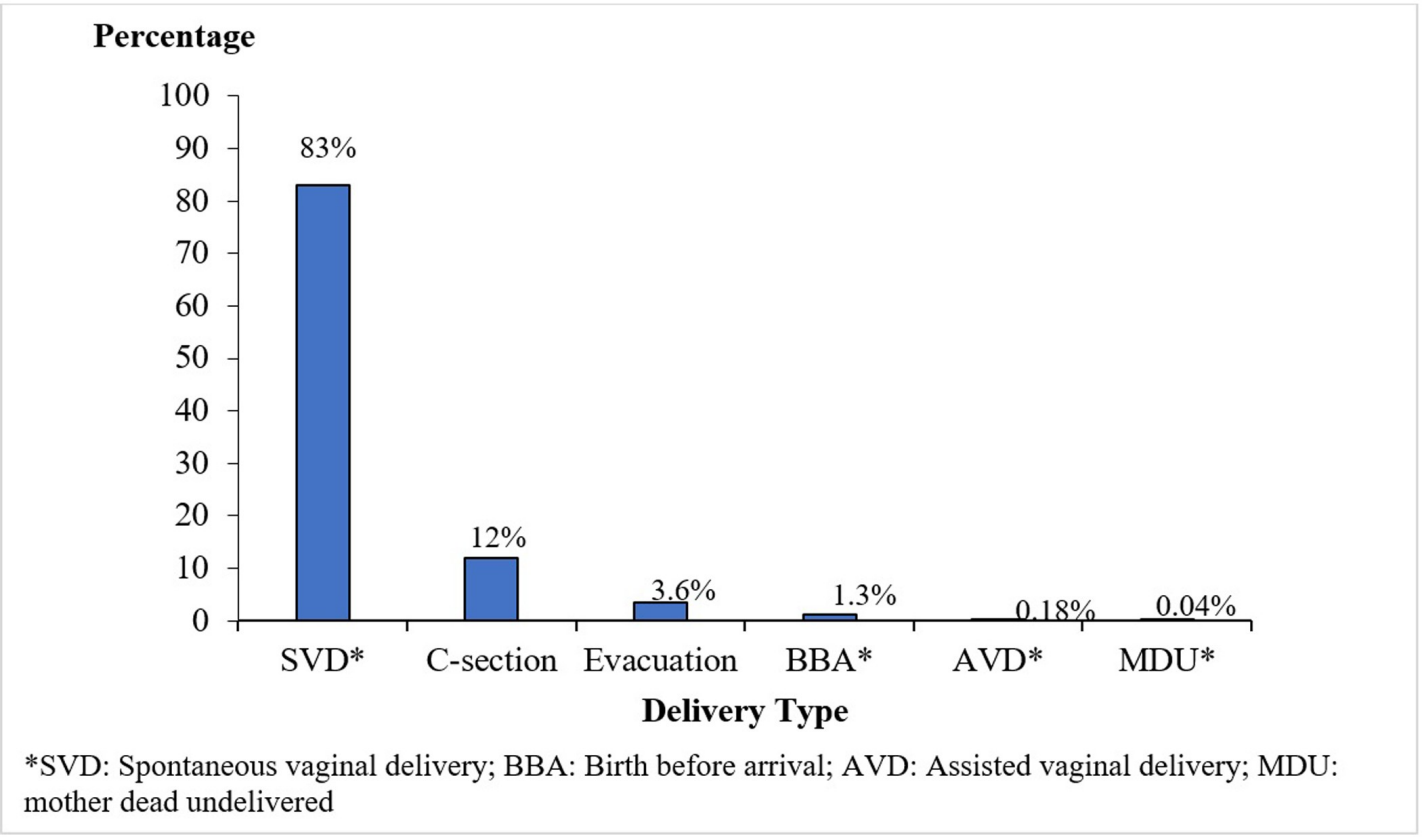
Types of delivery among pregnant women in the SMGL initiative in Western Uganda, 2015–2016.

For the 114,899 (99%) women for whom delivery type was recorded, 94,807 (83%) had a spontaneous vaginal delivery (SVD), 20,092 (17%) required an assisted delivery, 14,212 (12%) had a Cesarean section, and 108,887 (95%) received services for active management of the third stage of labor (Fig 3). The study data included 97 types of maternal complications. In total, 23,993 (21%) experienced ≥1 complication, including obstructed labor (3,621, 15%) and abortion (3,868, 3.2%).

### Associations between HIV status, ARV use and outcomes of women and newborns in the SMGL initiative

The risk of maternal death at SMGL sites was 3.6 times higher among pregnant women living with HIV than those who were HIV-negative (RR = 3.6, 95% CI = 2.4–5.5) (Table 3). Pregnant women living with HIV also had an increased risk of maternal sepsis (RR = 2.1, 95% CI = 1.3–3.3) and low birthweight for their infants (RR = 1.2, 95% CI = 1.1–1.3). However, being HIV-positive was mildly protective against having a maternal complication (RR = 0.95, 95% CI = 0.87–0.97) and perinatal death (RR = 0.78, 95% CI = 0.68–0.89) (Table 3).

**Table 3:** Associations between HIV status and maternal and perinatal or neonatal outcomes in the SMGL initiative in Western Uganda, 2015–2016.

Outcomes	HIV-positive n = 8,307 (%)	HIV-negative n = 99,339 (%)	RR (95% CI)
**Maternal outcome**			
Death	28 (0.34)	93 (0.094)	3.6 (2.4–5.5)*
Sepsis	19 (0.23)	111 (0.11)	2 (1.3–3.3)*
Anemia	10 (0.12)	98 (0.99)	1.2 (0.64–2.3)
Assisted delivery	1,071 (13)	12,616 (13)	1 (0.96–1.1)
Hemorrhage	122 (1.5)	1,458 (1.5)	1 (0.83–1.2)
Any complication	1,208 (15)	15,698 (16)	0.92 (0.87–0.97)*
**Perinatal or neonatal outcome**			
Low birth weight	697 (8.4)	7,212 (7.3)	1.18 (1.09–1.3)*
Neonatal death	55 (0.66)	723 (0.73)	0.91 (0.69–1.19)
Low Apgar score at 1min	555 (6.7)	8,381 (8.4)	0.81 (0.75–0.88)*
Fresh still birth	94 (1.1)	1,489 (1.5)	0.76 (0.61–0.93)*
Macerated still birth	76 (0.91)	1,199 (1.2)	0.76 (0.60–0.96)*
Low Apgar score at 5min	275 (3.3)	4,340 (4.4)	0.76 (0.6–0.87)*

*statistically significant association

Pregnant women living with HIV who did not use ARVs had an increased risk of having any complication (RR = 1.9, 95% CI = 1.6–2.2). This group also had a higher risk of maternal sepsis (RR = 19, 95% CI = 7.7–47), maternal death (RR = 15, 95% CI = 7.1–31), and their babies were twice as likely to die (RR = 2.3, 95% CI = 1.6–3.4) compared to pregnant women living with HIV who used ARVs (Table 4).

**Table 4:** Associations between ARV use and maternal and perinatal or neonatal outcomes among women living with HIV enrolled in SMGL initiative in Western Uganda, 2015–2016.

Outcomes among HIV positive	Didn’t use ARVs n = 561 (%)	Used ARVs n = 7,746 (%)	RR (95% CI)
Maternal sepsis	11 (1.9)	8 (0.10)	19 (7.7–47)*
Maternal death	14 (2.5)	14 (0.18)	15 (7.1–31)*
Maternal malaria	7 (1.2)	13 (0.17)	7.4 (2.9–19)*
Maternal anaemia	2 (0.36)	8 (0.10)	3.5 (0.73–16)
Perinatal death	30 (5.3)	194 (2.5)	2.3 (1.6–3.4)*
Neonatal death	7 (1.2)	48 (0.62)	2.1 (0.99–4.6)
Any maternal complication	146 (26)	1,062 (14)	1.9 (1.6–2.2)*
Low birth weight	55 (9.8)	642 (8.3)	1.3 (0.9–1.6)
Maternal hemorrhage	10 (1.8)	112 (1.4)	1.2 (0.65–2.3)

*statistically significant association

## Discussion

At health centres implementing the SMGL initiative that were evaluated in Uganda, common adverse outcomes among pregnant women included hemorrhage and sepsis; among newborns, common adverse outcomes included low Apgar scores and low birth weight. Being HIV positive and failing to use ARVs among the women who were HIV-positive increased the risk of adverse maternal and perinatal outcomes.

In our study, the MMR was 258 per 100,000, lower than the pre-SMGL (baseline) district-wide MMR of 452 per 100,000 in 2012 (P < 0.0001) and the national rate of 336 per 100,000 live births in 2016 (P = 0.0008) [12, 18, 26]. Similarly, the SMGL facility NMR of 7.6 per 1,000 was lower than the baseline pre-discharge NMR of 8.4 per 1,000 in 2012 (p = 0.04) and the national rate of 19 per 1,000 in 2016 (p<0.0001) [12, 18, 26]. The improvements in maternal and neonatal mortality rates reported by SMGL facilities indicate the potential of this intensive approach for lowering deaths among pregnant women and newborns in Uganda. When appropriately implemented, the SMGL approach can limit the risk of the leading causes of maternal deaths in Uganda, which are delays in seeking and accessing quality care to manage complications [17, 27]. The prevalence of anaemia at the time of delivery in this group was less than one percent. In contrast, a 2021 Ugandan metanalysis reported a prevalence of anaemia in pregnancy of 30% [28]. Although the Ministry of Health recommends the provision of iron and folic acid supplementation to pregnant women to prevent anemia, low adherence and drug shortages interfere with program effectiveness [29]. At SMGL facilities, pregnant women had access to supplements and close monitoring of anaemia, which could have contributed to the low prevalence. Through SMGL implementation, accessible lifesaving emergency care for pregnant women and optimal newborn care for newborns can further reduce complications arising during labor, delivery, and immediately after birth [17, 27].

The HIV prevalence in this group (7.2%) was comparable to the 2017 national prevalence of 7.6% among women [19]. Pregnant women living with HIV in our study had a higher risk of maternal sepsis, death, and of having babies with low birth weight. HIV infection in pregnancy is associated with altered immune responses in both mother and child and an increased risk of adverse outcomes [8, 30]. At health centres implementing SMGL, HIV testing and treatment were integrated into routine labour and delivery services [22], providing mothers who tested positive with opportunities for maternal care. This could potentially explain the reduced risk of maternal complication and of perinatal death among babies of women who were women observed in our study. However, further investigation would be needed to confirm this hypothesis.

ARV use in this group (94%) was comparable to the national ARV use rates among women who are HIV-positive (95%) [19]. In 2012, Uganda launched the Option B+ prevention of mother to child transmission (PMTCT), in which all HIV-positive pregnant women and their exposed babies received ARVs, regardless of their CD4 levels [19]. Furthermore, HIV testing and provision of ARVs was an integral component of the SMGL approach [22]. Although ARV use among women who were HIV-positive in our analysis was high, women who did not use ARVs had a significantly increased risk of specific complications, malaria, sepsis, and of having babies with low birthweight. HIV -induced immune suppression is linked to increased susceptibility to infections like tuberculosis, pneumonia, and sepsis during pregnancy and the postpartum period [8]. ARVs suppress viral replication, and can reduce susceptibility to infections that may affect maternal and perinatal outcomes [31]. Our findings are consistent with previous reports that preterm births, low birthweight, and still births are higher among HIV-infected non-ARV users than those using ARVs [7, 11].

Despite Uganda’s success in the implementation of PMTCT, challenges in wider access to and utilization of services remain. Delays to initiate and sustain antenatal care (ANC), long patient waiting times, HIV-related stigma, and transportation to the health facilities are some of the barriers that affect adherence [32, 33]. Even in health centres where quality obstetric and neonatal care and ARVs are accessible, such as in SMGL-implementing facilities, strengthening measures to promote adherence, such as the provision of spaces that are conducive for communication between health care providers and women who are HIV-positive, can improve attendance at antenatal care and potentially early enrolment on ARVs [32]. Research on innovative complementary services to encourage women who are HIV-positive to adhere to using ARVs could be useful. Our findings complement evidence that the SMGL approach provides life-saving health services for mothers and newborns [17].

## Limitations

The analysis was carried out using previously collected data, and therefore it was not possible to assess other factors that could affect maternal and perinatal outcomes like ANC attendance, partner support, transport or access to the health centre, and HIV viral load. The SMGL sites were purposively selected for the program implantation and this could limit the generalizability of the findings. The conclusions of the study are, however, based on a significant sample size of 116,066 women collected over a two-year period.

## Conclusion

Health facilities that implemented the SMGL initiative had lower MMR and NMR than national rates. There was an increased risk of maternal death, sepsis, and low birth weight among women who were HIV-positive. Not using ARVs increased the risk of all adverse maternal and perinatal outcomes. To some extent, lessons learnt from SMGL are being sustained in Uganda’s maternal health care system package [17]. Commitment to improving health care continues through improved policies and capacity building by the Ministry of Health; empowered community health workers continue to advocate for improved services at rural facilities [18]. In addition to sustaining the SMGL approach to improving maternal and newborn health services, strengthening care for pregnant women who are HIV-positive and their newborns in health centres in Uganda and developing additional approaches that can sustainably improve antiretroviral therapy enrolment and adherence among this group, even at facilities already equipped with maternal and newborn health services, may continue to reduce MMR and NMR.
